# Aberrant Regulation of the BST2 (Tetherin) Promoter Enhances Cell Proliferation and Apoptosis Evasion in High Grade Breast Cancer Cells

**DOI:** 10.1371/journal.pone.0067191

**Published:** 2013-06-20

**Authors:** Aejaz Sayeed, Gloria Luciani-Torres, Zhenhang Meng, James L. Bennington, Dan H. Moore, Shanaz H. Dairkee

**Affiliations:** California Pacific Medical Center Research Institute, San Francisco, California, United States of America; Wayne State University School of Medicine, United States of America

## Abstract

Normal cellular phenotypes that serve an oncogenic function during tumorigenesis are potential candidates for cancer targeting drugs. Within a subset of invasive primary breast carcinoma, we observed relatively abundant expression of Tetherin, a cell surface protein encoded by the *Bone Marrow Stromal Cell Antigen (BST2)* known to play an inhibitory role in viral release from infected immune cells of the host. Using breast cancer cell lines derived from low and intermediate histopathologic grade invasive primary tumors that maintain growth-suppressive TGFβ signaling, we demonstrate that BST2 is negatively regulated by the TGFβ axis in epithelial cells. Binding of the transcription factor AP2 to the *BST2* promoter was attenuated by inhibition of the TGFβ pathway thereby increasing BST2 expression in tumor cells. In contrast, inherent TGFβ resistance characteristic of high grade breast tumors is a key factor underlying compromised *BST2* regulation, and consequently its constitutive overexpression relative to non-malignant breast epithelium, and to most low and intermediate grade cancer cells. In both 2-dimensional and 3-dimensional growth conditions, *BST2*-silenced tumor cells displayed an enhancement in tamoxifen or staurosporine-induced apoptotic cell death together with a reduction in the S-phase fraction compared to BST2 overexpressing counterparts. In a subset of breast cancer patients treated with pro apoptotic hormonal therapy, *BST2* expression correlated with a trend for poor clinical outcome, further supporting its role in conferring an anti apoptotic phenotype. Similar to the effects of gene manipulation, declining levels of endogenous *BST2* induced by the phytoalexin – resveratrol, restored apoptotic function, and curbed cell proliferation. We provide evidence for a direct approach that diminishes aberrant BST2 expression in cancer cells as an early targeting strategy to assist in surmounting resistance to pro apoptotic therapies.

## Introduction

Cell-intrinsic defenses against invading pathogens are a relatively new discovery [1]. To assist the host’s immune system in controlling microbial spread during productive infection, these defenses function as ‘restriction factors’. Among these factors, the bone marrow stromal antigen 2 (BST2)/tetherin is known to dramatically reduce the release of the human immunodeficiency virus, as well as a range of other viruses from infected cells [2], [3] by directly tethering virions to cells [4]. Viral infection is generally accompanied by the induction of interferon in the blood of infected individuals, which in turn initiates a cascade of feedback loops, known to involve BST2 upregulation [5]. Various response elements in the *BST2* gene promoter suggest that inflammatory cytokines may be involved in regulating its expression. In blood derived human immune cells, BST2 activation and type I interferon (IFN) production appear to share the same triggers ensuring that BST2 expression, and its role in the IFN negative feedback loop are synchronized [6]. Strong evidence also suggests that a viral gene product - Vpu, counteracts the inhibitory effect of BST2 on virus release within the host [3], [7]. BST2 is a 30–36 kd protein comprised of a cytoplasmic N-terminal region, followed by a transmembrane domain, a coiled-coil extracellular domain, and a C-terminal glycosylphosphatidylinositol (GPI) anchor [8], which in the human protein may represent a second transmembrane segment rather than a GPI anchor [9]. BST2 is normally detected in dendritic cells, terminally differentiated B cells, and bone marrow stromal cells [10], and is overexpressed in some hematological cancers [11].

The role of BST2 in solid tumors is largely undetermined. Other than an association with a breast cancer xenograft metastatic to the bone [12], the regulation and function of this membrane-associated gene product in cancerous breast tissue remains unknown. In the search for functional phenotypes characteristic of clinically aggressive breast cancer, our approach is to compare these with indolent, or less aggressive breast tumors, whereby the differential of mitotic index that masks many distinctive cellular features, could be overridden. Other approaches, such as direct molecular phenotyping of tumors of varying histopathologic grade, a well-established indicator of clinical outcome and breast cancer prognostication, has served largely to confirm previously observed microscopic differences in proliferation status [13]. To provide fresh insights in this regard, we have developed and characterized a panel of 16 novel cell lines of varying histologic grade [14]–[16] that supplement routinely used breast cancer cell lines uniformly representative of high grade breast cancer. Genomic profiling of an expanded panel of breast cancer cell lines revealed *BST2* among the top 100 significant genes differentially expressed in high vs. low grade tumor derived cell cultures - all in the proliferative state [16]. Striking differences in TGFβ responsiveness were a key factor underlying differential expression of other high grade associated genes [17]. Here, we demonstrate a role for the TGFβ axis in *BST2* regulation, and the effects of its experimental manipulation on downstream functional endpoints of apoptosis induction, and cell proliferation. Our study identifies an underlying regulatory mechanism gone awry that activates BST2 in breast epithelial cells during tumorigenesis, and contributes to the functional hallmarks of cancer portrayed by tumor cells overexpressing this gene product. A pragmatic strategy used here for normalizing endogenous BST2 expression experimentally could be applied towards increasing the effectiveness of standard cancer therapeutics in patients.

## Materials and Methods

### Primary Breast Carcinoma Cell Culture

The development of low, intermediate, and high grade immortalized primary breast cancer cell lines has been previously described [14]–[15]. All tumor cell lines were routinely propagated in optimized MCDB170 medium supplemented with 2% fetal bovine serum [18]. Treatments included exposure to 4 ng/ml TGFβ1 (R&D Systems, Minneapolis, MN, USA), 20 µM TGFβ inhibitor - SB-431542 hydrate (Sigma, St. Louis, MO, USA), 5 µM tamoxifen (Sigma) or 20–100 µM resveratrol (Sigma).

### Immunolocalization

Archived blocks of formalin fixed primary breast cancer samples collected with the written informed consent of the donor were obtained from the Department of Pathology, and used with the approval of the California Pacific Medical Center IRB committee. Each sample was graded and reviewed by participating pathologists (ZM, JLB). Sections of tumor tissue arrays and individual tumor blocks were deparaffinized and used for immunoperoxidase localization of a rabbit anti human BST2 antibody (FabGennix, Frisco, TX, USA). Membrane/cytoplasmic staining of BST2 in greater than 30% tumor cells were scored as a positive result.

For immunocytochemistry on cell cultures, paraformaldehyde-fixed tumor cells were permeablized with Triton X-100, incubated with rabbit anti BST2 followed by Alexa Fluor® 488 anti rabbit (Life Technologies, Grand Island, NY, USA), counterstained with propidium iodide (PI), and analyzed by confocal microscopy. Images from all cell lines were acquired at a constant voltage gain setting of the detector channel used. Data acquisition for no antibody control, and anti BST2 stained samples was performed under identical conditions.

For Western blotting, whole cell lysates were resolved by SDS-PAGE on 12% gels, transferred to PVDF membranes and probed with rabbit anti BST2, or mouse anti tubulin (Abcam, Cambridge, MA, USA) followed by peroxidase-labeled secondary antibody (Sigma), and chemiluminescent detection.

### Quantitative Real Time PCR (QPCR)

RNA isolated with the RNeasy kit (Qiagen Valencia, CA, USA) was used to synthesize cDNA for relative transcript quantitation. *BST2, AP2,* and *STAT3* sequence specific primers were employed in QPCR assays by SYBR Green analysis. *ACTB* control was included in each PCR reaction. Relative expression of *BST2* in tumor cell lines was normalized against mean ΔCt values of short-term primary cultures of nonmalignant human breast epithelial cells (n = 3). Relative expression of target genes was calculated as previously described [17].

### Chromatin Immunoprecipitation (ChIP) Assay

ChIP reagents were used as per manufacturer’s instructions (Upstate Biotechnology, Lake Placid, NY, USA) with minor modifications. Tumor cells fixed with 1% paraformaldehyde were sonicated, followed by immunoprecipitation of DNA-AP2 complexes with anti AP2 (Santa Cruz Biotechnology, Santa Cruz, CA, USA). Normal rabbit IgG served as a negative control. After de-crosslinking and purification, DNA was subjected to PCR with AccuPrime™ Taq DNA Polymerase (Life Technologies) and oligonucleotides for putative AP2 binding regions in the *BST2* promoter. Input controls were included in PCR reactions. PCR products were resolved on 2% agarose gels and visualized by ethidium bromide staining. Oligonucleotide sequences used for PCR amplification of ChIP DNA are as follows:

BST2 Promoter B Forward:ACAGTTGGCTGGCACCCAGTT

BST2 Promoter B Reverse:GAGGGTGCTGGAATCTTCTACGG

### RNA Interference

For *BST2* silencing, tumor cells were transfected with siCONTROL non–targeting siRNA or a set of 4 human *BST2* specific siRNAs (Thermo Scientific) using Lipofectamine 2000 (Life Technologies).

### Cell Proliferation and Apoptosis

Growth potential was measured as colony counts 7-days after plating single cell suspensions in 3% Matrigel (BD Biosciences, San Jose, CA, USA). To determine S-phase distribution, cells were pulse labeled with 10 µM BrdU for 1 hr, and stained with anti BrdU (Santa Cruz Biotechnology), and FITC-conjugated secondary antibody (Life Technologies). Proliferating cells represented by FITC-stained nuclei were counted as a percentage of all nuclei over 10 microscopic fields. Similarly, percent apoptosis were measured by immunolocalization of anti cleaved caspase 3 (Cell Signaling Technology, Danvers, MA, USA) in untreated vs. tamoxifen, or staurosporine treated tumor cell populations.

### Cell Cycle Analysis

Cells exposed to 10 µM BrdU for 1 hr were fixed with 70% ethanol, incubated with anti BrdU and FITC conjugated secondary antibody, counterstained with PI, and analyzed by FACScan (BD Biosciences) using CellQuest software (BD Biosciences).

### Clinical Outcome Analysis

A publicly available primary breast cancer microarray dataset - GSE4922 [19] was used for evaluating clinical outcome of *BST2* overexpressing patient subsets. The Kaplan-Meier estimates were used to plot survival curves, and the p-value from a log-rank test used to determine the statistical significance of hazard ratios. Disease-free survival was defined as the time interval from surgery until the recurrence or last date of follow up. All survival statistics were performed in the R package.

## Results

### Differential Expression of BST2 in Primary Breast Cancer

**Figure 1:**
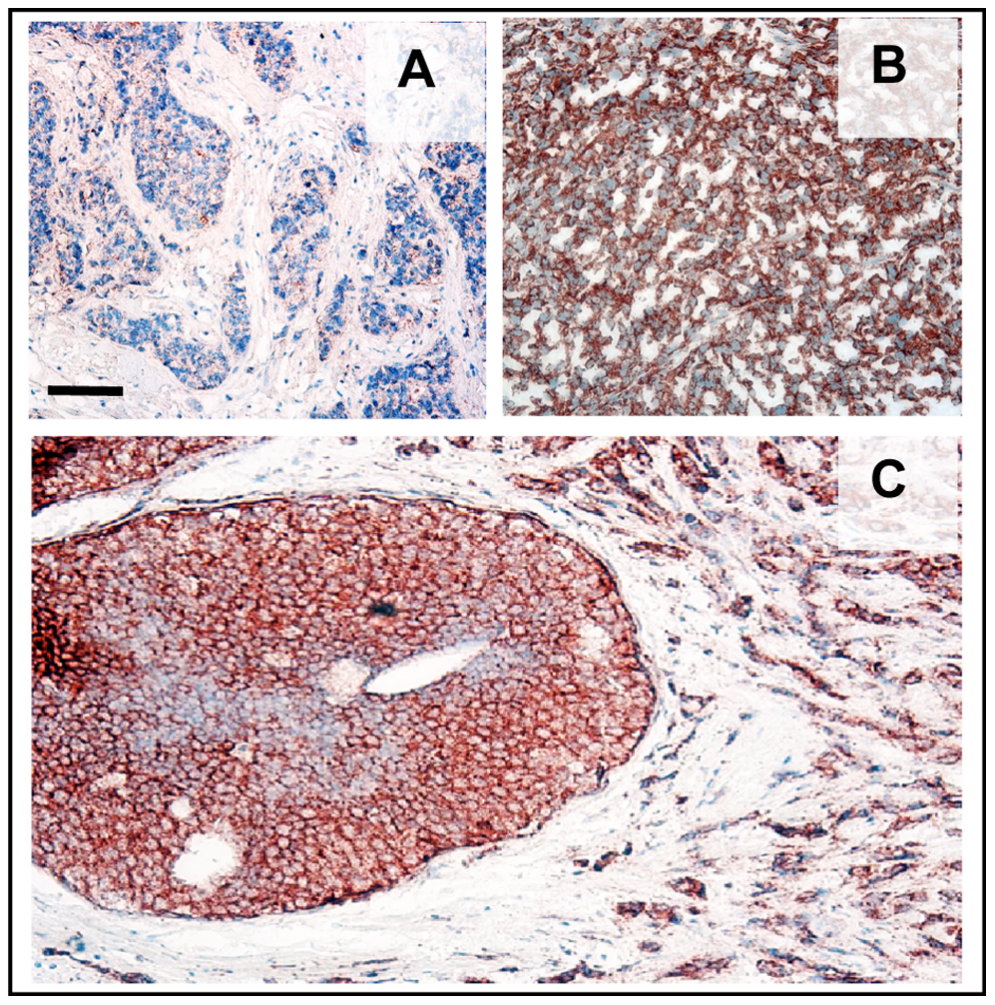
BST2 overexpression is associated with high histological grade of primary breast cancer. *****A.***** Low grade invasive primary breast tumor showing weak or no BST2 expressing cancer cells. Blue – hematoxylin counterstain. ***B.*** High grade invasive primary breast tumor displaying strong BST2 localization in cancer cells (brown). ***C.*** Homogeneous BST2 immunostaining in cells of coexisting ductal carcinoma *in situ* (DCIS), and invasive tumor. Bar –50 µm.

Immunoperoxidase localization of anti BST2 on 3 tissue arrays comprised of 234 primary breast tumor cores demonstrated strong membrane staining in the majority of intermediate and high grade tumors (grades 2 and 3, respectively), while minimal to no immunostaining was observed in low grade (grade 1) tumors (Table 1). BST2 immunolocalization in tissue arrays was verified in full-sized sections of tumor tissue blocks in several cases (Figure 1A–C). BST2 positive primary tumors with a concurrent DCIS component were found to display strong staining in both pre invasive and invasive tumor cells (Figure 1C).

**Table 1:** BST2 immunolocalization in primary invasive breast cancer.

Histological grade	Number of cases	Positive tumors (%)	Negative tumors (%)
1	30	8 (27)	22 (73)
2	120	103 (86)	17 (14)
3	134	126 (94)	8 (6)

The association between BST2 positive status and histological grade is significant (p<0.001).

**Figure 2:**
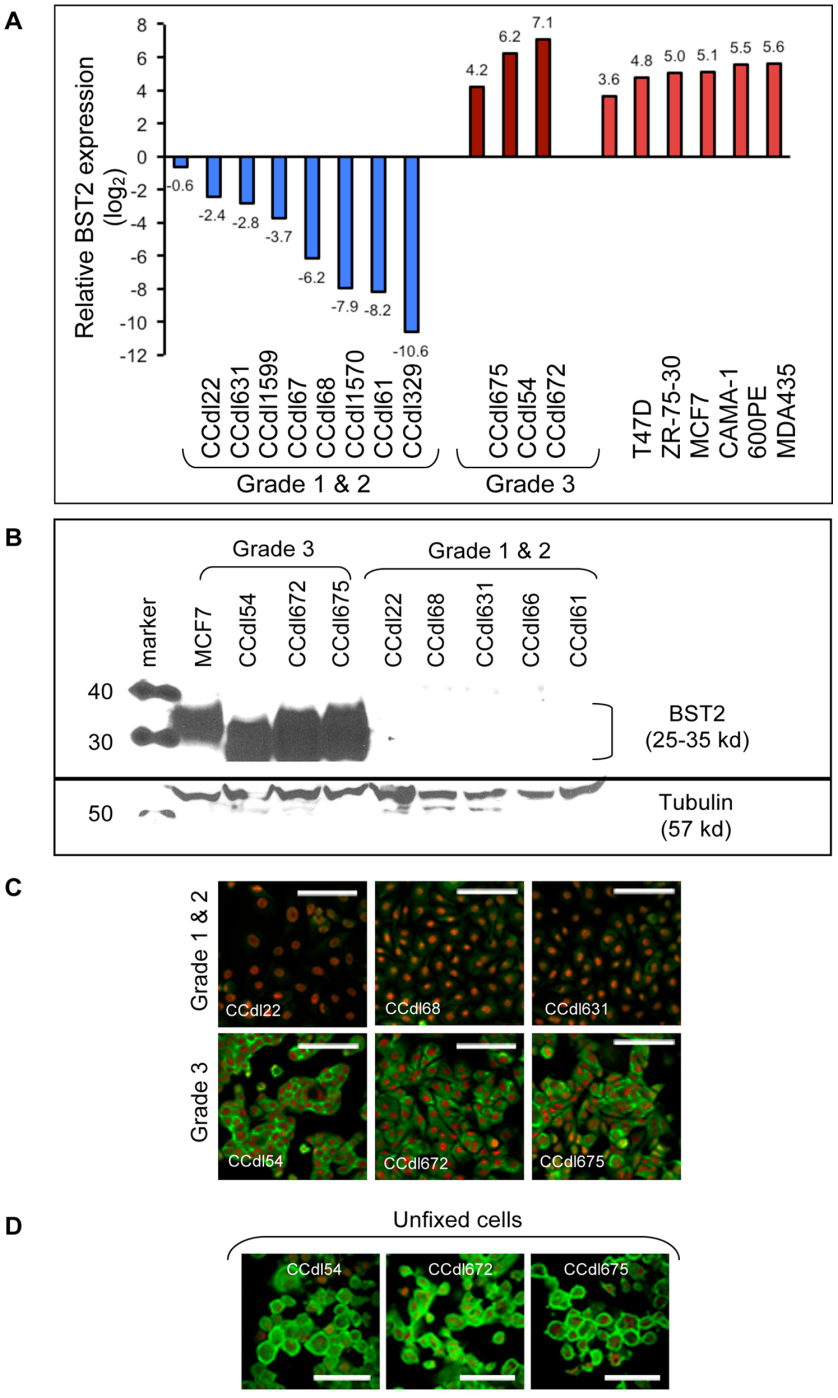
Differential BST2 expression in primary breast cancer of varying histological grade is maintained in tumor-derived cell lines. *****A***** *.* QPCR based *BST2* transcript levels in 17 breast cancer cell lines normalized to expression in non-malignant breast epithelium. ***B.*** Western blot analysis of BST2 protein (25–35 kd) in breast cancer cells. Tubulin used as a loading control. ***C.*** Microscopic images of BST2 immunostaining (green) in fixed, permeabilized breast cancer cells. Nuclei counterstained with propidium iodide (red). Bar –50 µm. ***D.*** BST2 immunolocalized at the cell membrane in live, unfixed breast cancer cells. Bar –25 µm.

In our previous expression profiling study of 14 novel primary breast cancer cell lines of varying histological grade (grade 1: n = 3; grade 2: n = 7; and grade 3: n = 4) developed in our laboratory, and 51 breast epithelial cell lines established by others [16], *BST2* was among the most highly expressed genes in grade 3 tumor lines identified by Significance Analysis of Microarrays (SAM). Here, we have established functional phenotypes resulting from differential BST2 expression by using breast cancer cell lines of varying grade. Grade associated variation in *BST2* transcript levels was first confirmed by QPCR analysis of breast cancer cell lines normalized to that of non-malignant primary breast epithelial cultures derived from reduction mammoplasty tissue (Figure 2A). Using a subset of 3 grade 3, and 5 grade 1 & 2 cell lines, differences in transcript levels were further confirmed by protein quantitation (Figure 2B), and by immunofluorescence microscopy (Figure 2C). Grade 3 tumor cell lines expressed abundant BST2 protein, whereas considerably lower or no protein expression was observed in grade 1 & 2 cell lines. Consistent with its localization in other cell types in various human tissues, BST2 immunostaining of unfixed live breast cancer cells displayed significant accumulation at the cell surface (Figure 2D).

### Negative Regulation of BST2 Expression by TGFβ

**Figure 3:**
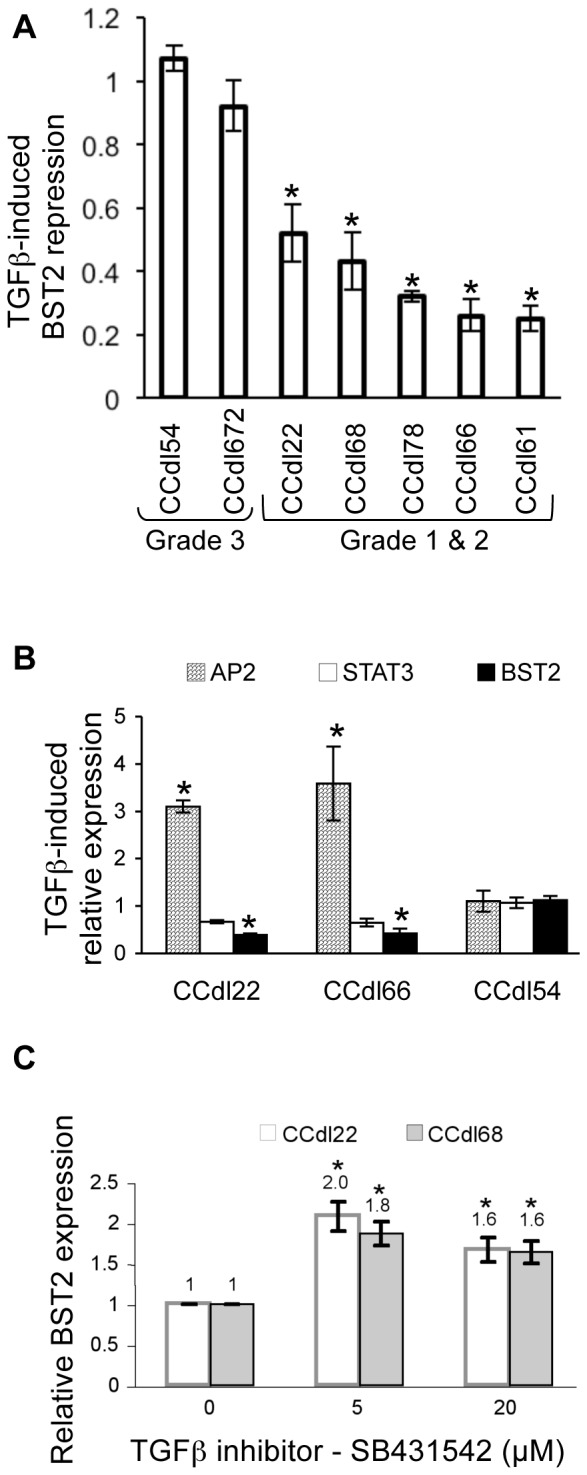
Regulation of *BST2* expression by TGFβ in breast cancer cells. ***A.*** Reduction in baseline *BST2* expression detected in 5 grade 1 & 2 cell lines after 24 hr TGFβ (4 ng/ml) treatment. Each QPCR reaction was carried out in triplicate, and values normalized to *ACTB* expression and to vehicle-only controls. Asterisks represent statistically significant differences (p<0.01) between untreated and TGFβ-treated samples. ***B.*** TGFβ-induced shift in *AP2* and *STAT3* transcript levels under the same treatment conditions employed in panel A. *BST2* reduction in grade 1 (CCdl22) & 2 (CCdl66) cell lines is accompanied by a decline in *STAT3* levels, while *AP2* transcripts increase significantly. No significant changes were observed in grade 3 (CCdl54) cells. Each assay was done in triplicate. Asterisks indicate a significant difference (p<0.01) between untreated and TGFβ-treated samples. ***C.*** Inhibition of the TGFβ pathway with 24 hr SB-431542 treatment increased *BST2* expression in grade 1 cell lines (CCdl22, CCdl68). Data acquired in triplicate shows significant differences (p<0.01) between test and vehicle-only cell samples, indicated by asterisks.

Exposure of grade 1 & 2 tumor cell lines to TGFβ for 24 hr resulted in further suppression of *BST2* transcripts whereas no change was observed in grade 3 cell lines by QPCR analysis (Figure 3A), suggesting transcriptional regulation of *BST2* in TGFβ responsive tumor cells. *BST2* reduction was accompanied by a significant (p<0.01) increase in expression of *AP2,* a transcription factor positively regulated by TGFβ. Additionally, expression of *STAT3,* a gene negatively regulated by TGFβ, was reduced under these conditions (Figure 3B). Transcriptional alterations of above mentioned test genes were not induced by TGFβ exposure of grade 3 cell lines. In contrast to these effects, treatment of 2 independent grade 1 tumor cell lines with the TGFβ inhibitor - SB431542, induced up to 2-fold increase in *BST2* (p<0.01) (Figure 3C).

### Site-specific Binding of AP2 to the BST2 Promoter

**Figure 4:**
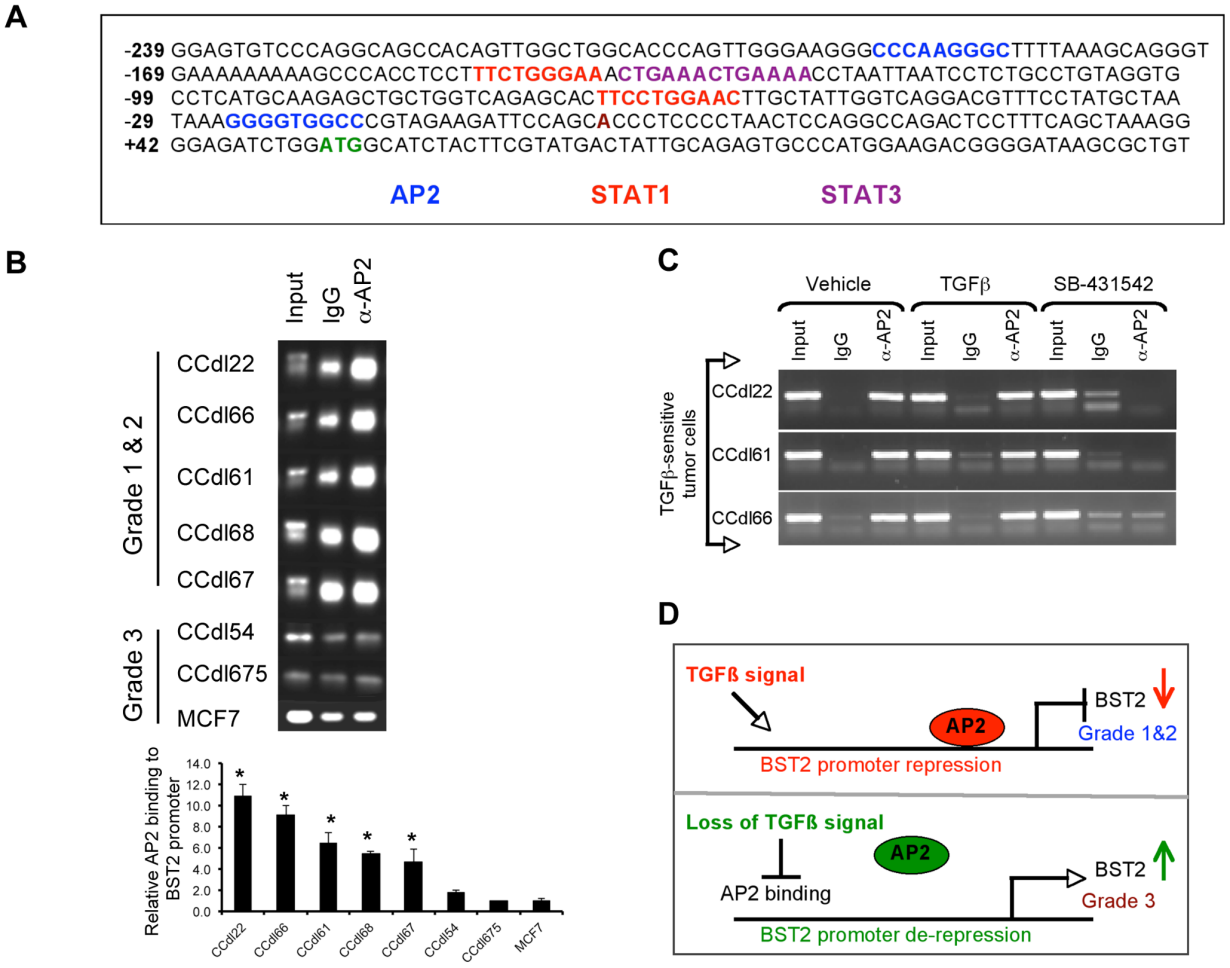
Differential AP2 binding to the *BST2* promoter in primary breast cancer cell lines of varying histologic grade. ***A.*** Schematic of the *BST2* promoter region spanning 239 bp upstream of the transcriptional start site, including 111 bp of exon 1. Numbering is relative to the translation start site, highlighted in green (+1). Potential *cis*-regulatory elements shown are either on the plus (+) or minus (–) DNA strands. Regulatory binding sequences: AP2 (blue), STAT1 (red) and STAT3 (purple). ***B.*** ***Top panel*** - Chromatin immunoprecipitation (ChIP) performed with anti AP2, or control IgG in 8 breast cancer cell lines. Significant AP2 recruitment to the *BST2* promoter observed only in grade 1 (CCdl22, CCdl68, CCdl67) & grade 2 (CCdl66, CCdl61) cell lines. ***Bottom panel*** **-** DNA from ChIP samples in top panel analyzed by QPCR. Primers encompassing putative AP2 binding sites from −221 to +6 were used. Melt curves were analyzed to ensure amplification of a single product. Each reaction was performed in triplicate. The plot represents relative binding efficiency determined by 2^-ΔΔ^C_T_, where ΔC_T_ is the difference between input C_T_ and immunoprecipitated C_T_; ΔΔC_T_ is the difference between AP2-immunoprecipitated ΔC_T_ and IgG-immunoprecipitated ΔC_T_. Asterisks represent statistical significance (p<0.01). ***C*** *.* Inhibition of AP2 binding to the *BST2* promoter in TGFβ-responsive primary breast cancer cell lines. Prior to processing for ChIP, cells were treated with vehicle, 4 ng/ml TGFβ, or 20 uM TGFβ inhibitor - SB-431542**.** Note striking reduction in AP2 binding in the presence of SB-431542 in 3 independent test cell lines. ***D.*** Hypothetical representation of *BST2* transcriptional regulation by the TGFβ axis. Intact TGFβ regulation mediated by AP2 binding to the *BST2* promoter enables maintenance of low baseline expression in grade 1 & 2 breast cancer cells.

Towards a direct analysis of *BST2* regulation, we first evaluated the 240 bp region of the human *BST2* promoter and exon 1 by Vector NTI Suite software and detected the presence of AP2 and STAT binding sites within this region (shown diagrammatically in Figure 4A). Since QPCR analysis showed more than 3-fold induction of *AP2* expression by TGFβ within responsive low grade tumor cells, whereas only marginal to no change was detected in *STAT3* (Figure 3B), and *STAT1* transcript levels (data not shown), thus further analysis of AP2 binding was prioritized. ChIP assays were carried out using anti AP2, and promoter-specific oligonucleotides. Strong AP2 binding to the *BST2* promoter was observed in 5/5 grade 1 & 2 tumor cell lines whereas no binding was detected in 2/2 grade 3 lines, and in MCF7 cells (Figure 4B, *top panel*). QPCR analysis of ChIP-derived DNA from all test samples confirmed gel based findings of AP2 recruitment to the *BST2* promoter (Figure 4B, *bottom panel*). Finally, a direct role for TGFβ in mediating AP2 binding to the *BST2* promoter was confirmed in 3 independent primary breast tumor lines treated with the TGFβ inhibitor - SB 431542 prior to ChIP analysis (Figure 4C). Baseline TGFβ levels (approximately 1 ng/ml in 2% serum supplemented growth media used here) sufficed to induce AP2 binding to the *BST2* promoter in 3/3 TGFβ responsive low/intermediate grade breast cancer cell lines. While TGFβ induction of *AP2* transcripts was observed by direct QPCR measurements (Figure 3C), this effect was not detectable by ChIP likely due to differences in assay sensitivity. However, AP2 binding to the *BST2* promoter was effectively disrupted in the presence of the TGFβ inhibitor in all test cell lines (Figure 4C). Taken together, these observations suggest a model wherein AP2 binding to the *BST2* promoter triggers gene suppression and serves as the basis of differential BST2 expression in low vs. high grade tumors (Figure 4D).

### Induction of Apoptosis and Inhibition of Cell Proliferation Promoted by Loss of BST2 Expression

**Figure 5:**
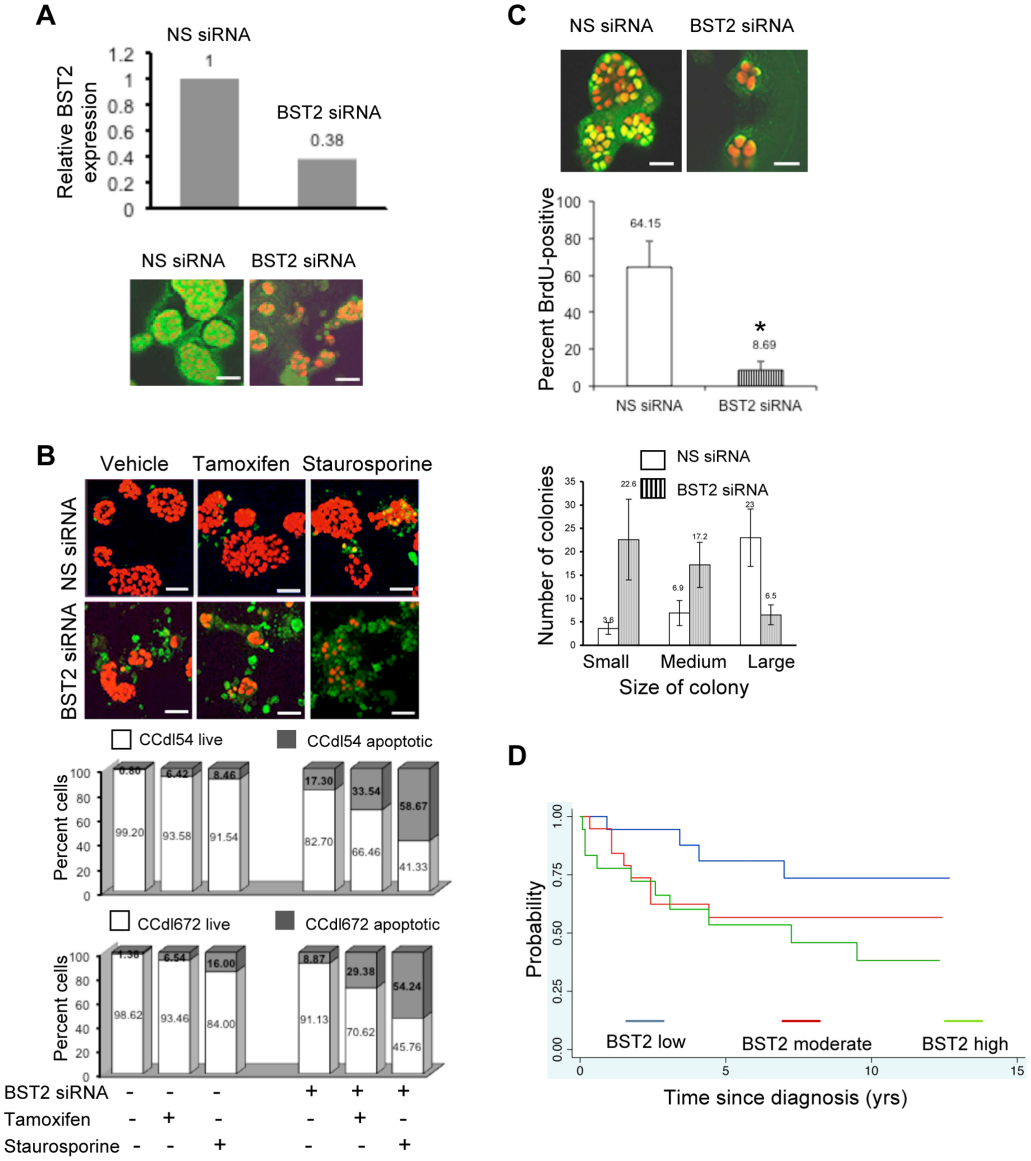
Functional consequences of endogenous BST2 overexpression in high grade primary breast cancer cells. ***A. Top panel*** – Reduction of *BST2* expression after transient knockdown by *BST2* siRNA transfection of grade 3 tumor cells (CCdl54). Values normalized to *ACTB* expression. ***Bottom panel*** – BST2 immunolocalization (green) on day-6 post knockdown, showing sustained reduction in *BST2* siRNA transfected cells plated in Matrigel**;** nuclei counterstained with PI (red). Bar - 50 µm. ***B*** *. BST2* knockdown enhances drug-mediated apoptotic cell death in 6-day old tumor cultures plated in Matrigel, and treated with 5 µM tamoxifen, or 5 µM staurosporine for 24 hrs. ***Top panel*** – immunolocalization of anti cleaved caspase 3 (green); nuclei counterstained with PI (red). Bar - 50 µm. ***Bottom panel*** – Data plotted as percent apoptotic cells in 2 independent BST2 overexpressing cell lines (CCdl54, CCdl672). Total number of PI-stained nuclei (>100), and proportion of cleaved caspase 3 positive tumor cells was averaged for 3 optical fields scanned with a 20× objective. Differences in apoptotic cell yield between NS siRNA vs. *BST2* siRNA-transfected cultures after treatment with each drug were significant (p<0.01). ***C*** *.* Growth reduction induced by *BST2* knockdown measured by BrdU incorporation in primary tumor cells plated in Matrigel. ***Top panel*** - BrdU immunolocalization (yellow) in tumor nuclei (CCdl54) counterstained with PI (red) 7-day post transfection with *BST2* siRNA. Bar - 25 µm. ***Middle panel*** - Data plots represent the fraction of BrdU positive proliferating cells in control (>100) and *BST2* knockdown cultures averaged from 5 optical fields scanned with a 20× objective. Asterisk denotes a significant (p<0.01) difference. ***Bottom panel*** – Data plots demonstrate an increase in the number of small (4-cell) and medium (8- to 12-cell) colonies accompanied by a decrease in the number of large-sized colonies (>25-cell) in *BST2* siRNA-treated Matrigel cultures. ***D.*** Stratification of hormone-treated ER+ breast cancer (n = 66) based on *BST2* transcript levels. The Kaplan Meier plot suggests a trend whereby a relatively poor clinical outcome is conferred upon cases with moderate (red) or high (green) *BST2* expression compared to those with low gene expression (blue).

To evaluate its functional role in breast cancer, BST2 overexpressing cells were transfected with *BST2* siRNA and evaluated as 3-dimensional Matrigel cultures. Reduction in BST2 expression was confirmed both by QPCR and immunofluorescence (Figure 5A). As measured by immunofluorescence localization of anti cleaved caspase 3, a decline in BST2 levels detectably enhanced apoptotic cell death induced by 2 independent pro apoptotic drugs - 4-hydoxy tamoxifen or staurosporine (Figure 5B).

Reduced *BST2* transcript levels also led to decreased cell proliferation measured by BrdU incorporation and immunofluorescence localization of anti BrdU (Figure 5C, *top panel)*. A decline in anti BrdU-stained cell populations (Figure 5C, *middle panel)* was accompanied by smaller colony size in BST2 overexpressing cell lines transfected with *BST2* siRNA vs. nonspecific siRNA controls (p<0.01) (Figure 5C, *bottom panel*).

### Association of BST2 Expression with Clinical Outcome of Breast Cancer Patients

Based on the demonstrated role for BST2 in apoptosis evasion *in vitro*, we used the GSE4922 gene expression dataset (http://www.ncbi.nlm.nih.gov/geo/) to explore *BST2* stratification potential in breast cancer patients treated with pro apoptotic hormonal therapy. This dataset includes a total of 66 ER+ patients who received single agent hormone treatment. *BST2* transcript values in these cases ranged from 6.4 to 10.3 relative units demonstrating that expression of this gene is variable in ER+ tumors. Using Martingale residuals as a measure of the influence of *BST2* expression on the hazard ratio in a Cox Proportional hazards model, an increasing hazard ratio was noted for 55 cases where *BST2* expression in the tumor ranged between 7.0 to 9.5 units. Twenty-two of these cases recurred within 9.5 yrs. The risk of recurrence doubled for each unit increase in *BST2* expression (HR 2.00, CI: 0.95–4.26, p = 0.07). Other prognostic parameters, such as: histologic grade (HR 1.85, CI: 0.86–3.95, p = 0.11), age at diagnosis (HR 0.99, CI: 0.95–1.04, p = 0.96), tumor size (HR 1.01, CI: 0.97–1.05, p = 0.54), and lymph node status (HR 1.36, CI: 0.53–3.49, p = 0.51) were less or not predictive of outcome. Based on these findings, data were divided into increasing *BST2* expression tertiles, and a Kaplan-Meier plot was generated (Figure 5D). A trend was evident, whereby patients in the top 2 tertiles of *BST2* expression showed a shorter interval of disease-free survival compared to those with lower *BST2* expression in the primary tumor (p = 0.12), supporting our *in vitro* molecular and functional studies on breast cancer cell lines. The predicted trend for hormonal therapy response observed with *BST2* as a single gene classifier was consistent with the grade gene index (GGI)-predicted profile in this subgroup of ER+, hormone-resistant patients (HR 2.9, CI: 1.26–6.95, p = 0.01). However, unlike *BST2* alone, the GGI predictor uses a multigene panel.

### Resveratrol Mediated Abrogation of Endogenous BST2 Expression

**Figure 6:**
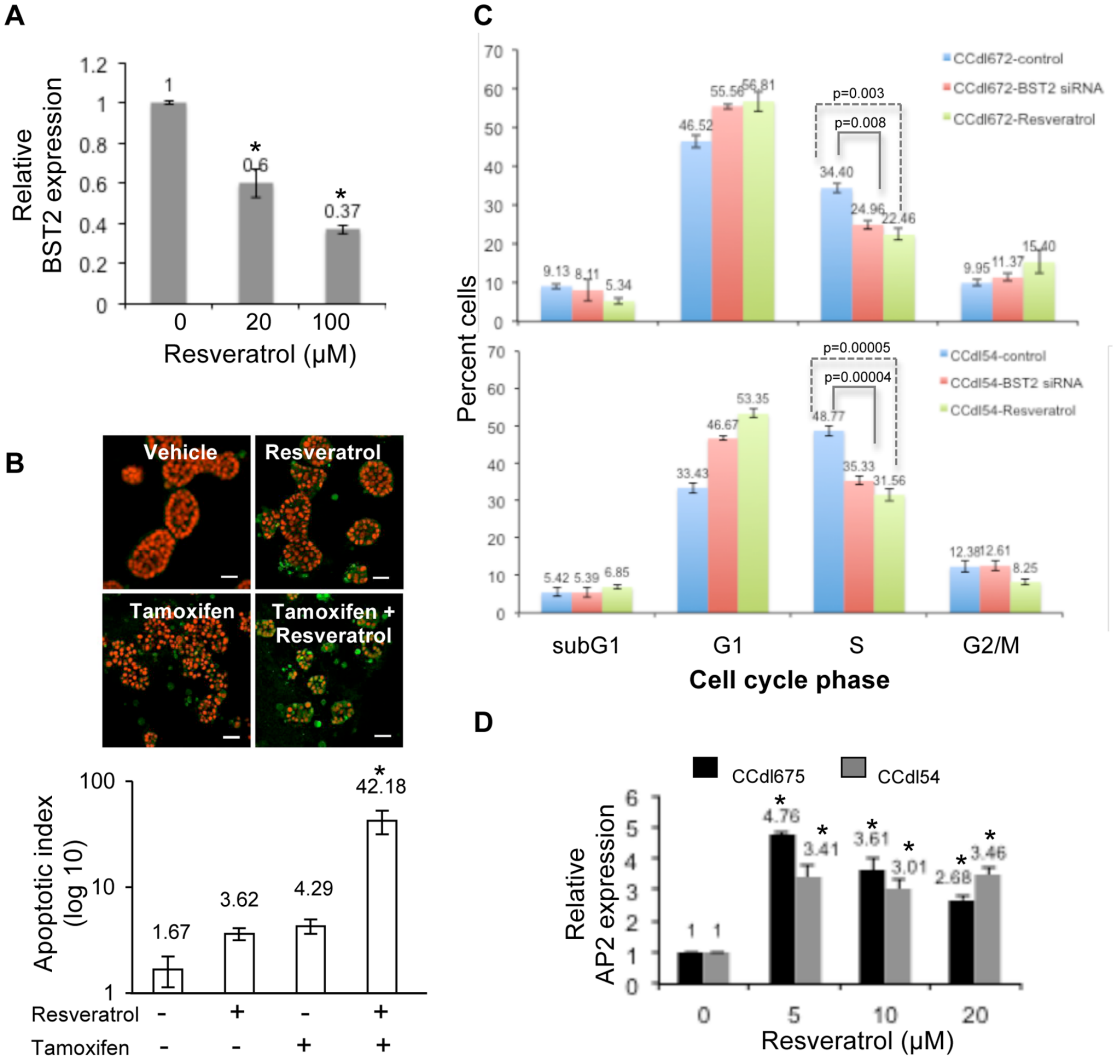
Resveratrol attenuates endogenous *BST2* expression and disrupts downstream functional effects in high grade breast cancer cells. ***A.*** Tumor cells (CCdl54) treated with resveratrol for 24 hrs displayed a significant decline in *BST2* expression denoted by asterisks (p<0.01). QPCR analysis performed in triplicate, and normalized to *ACTB* expression. ***B.*** Enhancement of apoptotic cell death induced by 24 hr tamoxifen treatment of 6-day resveratrol (20 µM) supplemented tumor cultures plated in Matrigel. ***Top panel*** – immunolocalization of cleaved caspase 3 (green) in apoptotic cells; nuclei counterstained with PI (red). Bar - 25 µm. ***Bottom panel*** – Immunostaining data plotted as apoptotic index represents proportion of PI-stained nuclei (>100) vs. immunopositive cells in multiple microscopic fields scanned with a 20× objective, and averaged. Asterisk denotes significant (p<0.01) differences in the apoptotic index in the presence of tamoxifen alone vs. resveratrol pretreatment followed by tamoxifen. ***C.*** Resveratrol-mediated suppression of the cell cycle in high grade tumor cells (CCdl54, CCdl672) is similar to the effects of *BST2* silencing. A statistically significant reduction in S-phase resulting from both approaches for *BST2* attenuation is accompanied by an increase in the G1-fraction in independent cell lines. The data derived from BrdU labeling of 2 independent cell lines represents an average of 2 separate FACS runs on each cell line, with each treatment performed in triplicate sets. ***D.*** Induction of *AP2* expression in high grade tumor cells (CCdl675, CCdl54) treated with increasing concentrations of resveratrol. QPCR analysis performed in triplicate, and normalized to *ACTB* expression. Asterisks indicate significant differences (p<0.01) between control and treated samples.

Resveratrol is known to modulate AP2 binding within promoters of putative oncogenes, and included in over 60 human trials for a variety of diseases (clinicaltrials.gov). As a pragmatic approach for reversing gene expression in the clinical setting, we tested the *in vitro* effects of resveratrol on BST2. Overexpressing tumor cultures treated with increasing doses of resveratrol for 24 hr demonstrated a significant reduction in *BST2* transcript levels (p<0.01) (Figure 6A). In 3-dimensional Matrigel cultures treated with resveratrol, this change in expression effectively reproduced the downstream pro apoptotic effects of gene silencing observed with *BST2* siRNA transfection (Figure 6B). The synergistic effect of concurrent exposure to resveratrol and tamoxifen on apoptosis induction measured by anti cleaved caspase 3 immunolocalization was over 10-fold that of either treatment alone (p<0.01). Closely analogous to the consequences of *BST2* knockdown on the cell cycle, resveratrol treatment of tumor cultures induced a marked decline in S-phase cells accompanied by an increase in the G1 fraction (Figure 6C).

To evaluate whether BST2 attenuation was mediated by the mode of promoter regulation characteristic of TGFβ responsive tumor cells described above, *AP2* expression was measured in resveratrol-treated cells. Accompanying the suppressive effects on BST2 expression, increasing concentrations of resveratrol led to markedly increased *AP2* levels in 2 independent grade 3 cell lines (Figure 6D). This suggests the possibility of resveratrol induced *BST2* promoter repression by AP2 in TGFβ-resistant cells analogous to that occurring normally in TGFβ-responsive breast cancer cells.

## Discussion

BST2 overexpression in cancerous breast tissue specimens demonstrated here poses critical questions regarding usurpation of its normal host protective function through aberrant gene regulation. We have defined an oncogenic framework for *BST2* in breast cancer by correlative and functional analysis of clinical tissue and novel *in vitro* model systems, respectively. We show that the overexpression of this gene is characteristic of high histopathologic grade tumors known to display a higher mitotic index, and greater clinical aggressiveness. Generally, most high grade (poorly differentiated or grade 3) breast cancer is associated with poor clinical outcome. Aberrant signaling pathways that undermine the histological differentiation of such cancer cells could shed light on the functional underpinnings of breast cancer aggressiveness, and its timely control. We describe approaches and consequences of *in vitro* manipulation of *BST2* towards the overall goal of suppressing functional hallmarks of tumor aggressiveness.

Critical roles for BST2 have been previously established in the innate immune response. While its expression is induced by interferon (IFN) in the event of initial viral infection, BST2 itself negatively regulates IFN to minimize undesirable effects of prolonged IFN exposure, such as autoimmune diseases [20]. In terms of tissue distribution, it appears that different Transcription Factor Binding Sequences in the *BST2* promoter region might be activated in a cell type specific manner [6]. In dendritic cells, interaction of BST2 with its cognate receptor, immunoglobulin-like transcript 7 (ILT7) provides the negative signal to control IFN release [21]. As we have shown here, BST2 regulatory mechanisms in breast epithelium appear to be distinctive from those of immune cell types. Likewise, paracrine signaling phenotypes are known to be remarkably different from tissue to tissue. Genomic profiling of stromal-epithelial crosstalk identifying the signature of fibroblast triggered expression in tumor (FTExT) cells of the breast [22] is in stark contrast to that between bone marrow-derived fibroblasts and lymphoma cells [23].

Here, we have uncovered a causal link between the disruption of TGFβ signaling in high grade breast cancer and the constitutive activation of *BST2*. Our data suggest that aberrant BST2 overexpression promotes the consequences of obliterated TGFβ-mediated tumor suppressive effects in breast cancer, and subsequent loss of differentiation programs. Members of the TGFβ family are pluripotent cytokines exerting an array of biologic effects in a wide variety of cell types. Several studies have implicated the TGFβ pathway in the pathophysiology of cancer [24]. We show that the transcription factor, AP2 is a critical component of TGFβ-mediated regulation of *BST2* in breast cancer. Our data suggests that impaired TGFβ signaling leads to the inability of AP2 to bind the *BST2* promoter resulting in failed repression of this putative oncogene in high grade tumor cells. The role of AP2 in TGFβ-mediated signaling has been suggested by other studies [25]. Closely parallel to our observations of the negative regulation of *BST2* in breast cancer, AP2 is involved in the repression of promoter sequences of the oncogene, *ERBB2* [26], evident as an inverse correlation between AP2 and ERBB2 immunolocalization in archival tumor specimens [27]. In the latter study, AP2 positive tumors displayed a reduced mitotic index and a lower tumor grade, consistent with our findings of intact AP2 regulation of *BST2* in low grade breast cancer.

Predictive markers of therapeutic drug resistance are of significant utility towards the consideration of alternate treatment approaches in the clinic but rarely available. Antibody based targeting of BST2 upregulation in B cell lymphoma lesions delayed tumor growth [28] supporting our observations of a BST2-mediated role in breast cancer outcome. Although the TGFβ signaling axis has been implicated in endocrine response [29], molecular mediators have not been clearly identified. BST2 overexpression could serve to identify patients likely to relapse after pro apoptotic cancer therapies. Our data demonstrates a direct functional role of BST2 in the establishment of therapeutic resistance to an ER antagonist. Among changes in the expression of several other genes, profiling studies of tamoxifen-treated normal human mammary epithelial cell cultures have noted induction of *BST2* [30]. Further work is needed to decipher the molecular mechanism of BST2-mediated drug resistance, for example how BST2 interacts with, and influences the ER signaling cascade, could be illuminating. Our findings suggest the possibility of engaging resveratrol treatment as a long-term preventive modality, and as an adjunct to conventional therapeutic strategies, particularly since the anti carcinogenic effects of resveratrol are well known. At the molecular level, resveratrol is involved in the inhibition of oncogenic processes, including increased oxidoreductase activity, and oncogenic phenotypes such as BCL2, AKT, FAK, NF-kB, MMP9, and positive cell cycle regulators [31]. Specifically in the context of breast cancer, resveratrol inhibits TGFα resulting in the elevated expression of TGFβ2 [32]. Resveratrol mediated BST2 repression in the model systems studied here can be attributed to the induction of TGFβ regulated AP2. AP2 binding regions within promoters of putative oncogenes, such as *Na(+1)/H(+1) exchanger-1* (*NHE-1*), were previously identified targets of resveratrol [33].

The cellular models used here define an important role for the TGFβ axis as a major contributor to the well-known biological and clinical heterogeneity of breast cancer. Generally, in breast tumors classified as grade 1, and in a proportion of grade 2 cases, TGFβ acts as a potent tumor suppressor by repressing putative oncogenes [17]. In grade 3 and some grade 2 breast tumors, impairment of TGFβ-mediated signaling intermediates, often due to inactivating mutations [34], results in the derepression of these oncogenes, enabling an escape from TGFβ-dependent growth modulation, and culminating in an overall aggressive tumor phenotype. For direct cancer targeting, cell surface markers, such as BST2, are the proverbial “silver bullet” particularly if overexpressed in tumors prone to therapeutic resistance. Breast cancer therapies that target cell surface proteins, such as EGFR and ERBB2 are rare examples of successful biologically informed cancer treatments. Overall, BST2 is a strong candidate for consideration as a theranostic in the target discovery pipeline for breast cancer.
